# A Novel Truncated CHAP Modular Endolysin, CHAP^SAP26^-161, That Lyses *Staphylococcus aureus*, *Acinetobacter baumannii*, and *Clostridioides difficile*, and Exhibits Therapeutic Effects in a Mouse Model of *A. baumannii* Infection

**DOI:** 10.4014/jmb.2402.02042

**Published:** 2024-06-17

**Authors:** Yoon-Jung Choi, Shukho Kim, Ram Hari Dahal, Jungmin Kim

**Affiliations:** Department of Microbiology, School of Medicine, Kyungpook National University, Daegu 41566, Republic of Korea

**Keywords:** Endolysin, deletion mutants, resistance, *Acinetobacter baumannii*, *Staphylococcus aureus*

## Abstract

Development of novel antibacterial agents is imperative due to the increasing threat of antibiotic-resistant pathogens. This study aimed to develop the enhanced antibacterial activity and in-vivo efficacy of a novel truncated endolysin, CHAP^SAP26^-161, derived from the endolysin LysSAP26, against multidrug-resistant bacteria. CHAP^SAP26^-161 exhibited higher protein purification efficiency in *E. coli* and antibacterial activity than LysSAP26. Moreover, CHAP^SAP26^-161 showed the higher lytic activity against *A. baumannii* with minimal bactericidal concentrations (MBCs) of 5–10 μg/ml, followed by *Staphylococcus aureus* with MBCs of 10–25 μg/ml. Interestingly, CHAP^SAP26^-161 could lyse anaerobic bacteria, such as *Clostridioides difficile*, with MBCs of 25–50 μg/ml. At pH 4–8 and temperatures of 4°C–45°C, CHAP^SAP26^-161 maintained antibacterial activity without remarkable difference. The lytic activity of CHAP^SAP26^-161 was increased with Zn^2+^. In vivo tests demonstrated the therapeutic effects of CHAP^SAP26^-161 in murine systemic *A. baumannii* infection model. In conclusion, CHAP^SAP26^-161, a truncated endolysin that retains only the CHAP domain from LysSAP26, demonstrated enhanced protein purification efficiency and antibacterial activity compared to LysSAP26. It further displayed broad-spectrum antibacterial effects against *S. aureus*, *A. baumannii*, and *C. difficile*. Our in vitro and in-vivo results of CHAP^SAP26^-161 highlights its promise as an innovative therapeutic option against those bacteria with multiple antibiotic resistance.

## Introduction

The increasing prevalence of antibiotic-resistant bacteria, including multidrug-resistant (MDR), extensively drug-resistant (XDR), and pandrug-resistant (PDR) strains, presents a tremendous challenge to modern medicine [1-5]. Therefore, there is an urgent need to develop alternative therapeutic strategies to combat these pathogens. Bacteriophages (phages) and their peptidoglycan-degrading endolysins have emerged as promising candidates [6-9]. Endolysins, hydrolytic enzymes produced by phages’ genomes, demonstrate a broader host spectrum than their phages and have the advantages of rapid bacterial cell lysis, low risk of resistance development, and efficacy against biofilms and mucosal surfaces [10-17]. The structural diversity of endolysins contributes significantly to their functionality [18, 19]. The catalytic domains of enzymes, including amidases, glycosylases, endo-beta-N-acetylglucosaminidases, and CHAP (Cysteine, Histidine-dependent Amidohydrolases/Peptidases) domains, are designed to break specific peptidoglycan bonds within the bacterial cell wall [20-22]. Moreover, some endolysins often feature a binding domain that facilitates their specific binding to the bacterial cell walls, enhancing the antibacterial action [20, 23-27]. SH3b domain, one of the binding domains, that recognizes and binds to specific peptidoglycan cross-linked areas, ensuring the catalytic activity occurs precisely where needed [23]. Some endolysins also possess translocation domains that are essential for targeting Gram-negative bacteria because they help them cross the outer membrane barrier that characterizes Gram-negative bacteria [20, 21]. Endolysins can be categorized based on their structural composition into modular and globular endolysins [13, 21]. Modular endolysins consist of multiple functional domains that collaborate to enhance the enzymés effectiveness. This modular configuration allows for a flexible approach to target various bacterial cell wall structures and enables customization to improve specificity and efficiency against particular bacterial species. Conversely, globular (single-domain) endolysins comprise only one functional domain, usually catalytic, and lack separate binding module(s). Although their simple structure and lack of modular diversity may limit their range of activity, single-domain endolysins can be highly effective against specific targets and are optimized for distinct actions [13].

In our previous study, we reported on LysSAP26, an endolysin isolated from *Staphylococcus aureus* phage SAP26, which consists of 251 amino acids with a CHAP domain (20-109 amino acids) and an unidentified functional domain (110-251 amino acids) [28, 29]. LysSAP26 is able to inhibit not only Gram-positive bacteria, such as *S. aureus* and *Enterococcus faecalis*, but also Gram-negative bacteria, including *Pseudomonas aeruginosa*, *Klebsiella pneumoniae*, and *Acinetobacter baumannii* [29]. The current study aims to enhance the antibacterial activity with more protein purification yield of LysSAP26 by creating deletion mutants. By truncating the C-terminal region of LysSAP26, we produced two deletion mutants, CHAP^SAP26^-139 and CHAP^SAP26^-161, and assessed their antibacterial activities compared to the original enzyme.

CHAP^SAP26^-161 showed better antibacterial activities with higher protein yield than wild type enzyme. Our study presents that the CHAP domain of LysSAP26 can be promising antibacterial agent in treating multidrug-resistant bacterial infections and a candidate module for further endolysin engineering.

## Materials and Methods

### Bacterial Strains and Culture Conditions

A total of 96 MDR clinical isolates, including *S. aureus* (20), *A. baumannii* (17), *Clostridioides difficile* (3), *Enterococcus faecium* (20), *Klebsiella pneumoniae* (20), *Escherichia coli* (20), *Pseudomonas aeruginosa* (20), *Enterococcus faecalis* (20), and other strains, was acquired from the Kyungpook National University Hospital Culture Collection for Pathogens. In addition, reference strains were acquired from the Korean Collection for Type Cultures and American Type Culture Collection (ATCC). The bacteria were cultured in the media, such as Mueller–Hinton broth (DB, USA), Mueller–Hinton agar (MHA, USA), brain heart infusion (BHI) broth, and blood agar plates, and incubated for 24–48 h at 37°C. Under anaerobic conditions (5% H2, 5% CO_2_, and 90% N_2_) for *C. difficile* isolates, they were cultured using an anaerobic chamber [30, 31]. For the long-term storage of the bacterial strains, bacteria were kept in BHI broth containing 15% glycerol (v/v) and stored in -70°C.

### Construction of Truncated Endolysin-Expression Vectors

The LysSAP26 sequence in the SAP26 phage genome was used as a template to generate protein expression vectors for CHAP^SAP26^-139 and CHAP^SAP26^-161. PCR amplification involved specific primers (Table S1) and ExTaq Polymerase (Takara, Japan) [29, 32-35]. The PCR products were then purified using a clean-up kit (GeneAll, Republic of Korea) and digested with NdeI and XhoI at 37°C for 1 h. The digested products were ligated into the pET-21a (+) vector using T4 DNA ligase at 18°C for 3 h. The ligation product was transformed into *E. coli* DH5α, and the transformed bacteria were selected on Luria–Bertani (LB) medium (BioShop, Canada) agar plates containing ampicillin (150 μg/ml). Colony PCR was conducted by amplifying the insert to confirm successful cloning using the nde1-SAPlys primer and the T7 terminator primer [29, 32-35]. Finally, the PCR products were sequenced to confirm the accuracy of the constructed vector.

### Expression and Purification of Wild Type and Two Truncated Endolysins

Following the protocol previously reported by Kim *et al*., each of the LysSAP26 (753 bp), CHAP^SAP26^-139 (417 bp), and CHAP^SAP26^-161 (483 bp) expression vectors was transformed into *E. coli* BL21 (DE3) Star cells [29, 35]. Subsequent cultivation involved incubating the transformed cells in 1 L of LB medium supplemented with 150 μg/ml ampicillin at 37°C with a shaking speed of 150 rpm. This incubation continued until the optical density (OD) at 600 nm reached 0.5. Then, isopropyl β-D-1-thiogalactopyranoside (IPTG) was added to be 0.1 mM , and the culture was incubated further at 18°C for 16 h. After the incubation, cells were harvested by centrifugation, resuspended in lysis buffer (50 mM Tris-HCl, 500 mM NaCl, and 2% 2-mercaptoethanol; pH 7.4), and subsequently lysed using ultrasonication (Branson 450, USA). After centrifugation at 4°C, the supernatants were loaded onto a His-trap column (GE Healthcare, USA) that was installed in a fast protein liquid chromatography AKTA Prime PLUS chromatography system (Pharmacia, USA). Finally, the recombinant protein was purified using a 5-ml His-trap affinity column (GE Healthcare) with a 150 mM imidazole buffer. To eliminate residual imidazole from the purified fractions, dialysis was performed against the dialysis buffer (50 mM Tris-HCl, 500 mM NaCl, and 1 mM ZnCl_2_; pH 7.4). The purity and molecular weights of the proteins were verified by sodium dodecyl sulfate-polyacrylamide gel electrophoresis (SDS-PAGE) and western blot analysis. For the western analysis, an anti-His-Tag monoclonal antibody (Ab Frontier, Republic of Korea) was used as the primary antibody to detect His-tagged proteins through western blotting and a horseradish peroxidase-conjugated polyclonal rabbit antimouse immunoglobulin G (Dako, Denmark) was used as the secondary antibody. The concentration of the purified proteins was quantified using the Pierce BCA Protein Assay Kit (Thermo Fisher Scientific, USA) as described previously [29, 35].

### *In silico* Analysis of the Protein Structures

**Fig. 1:**
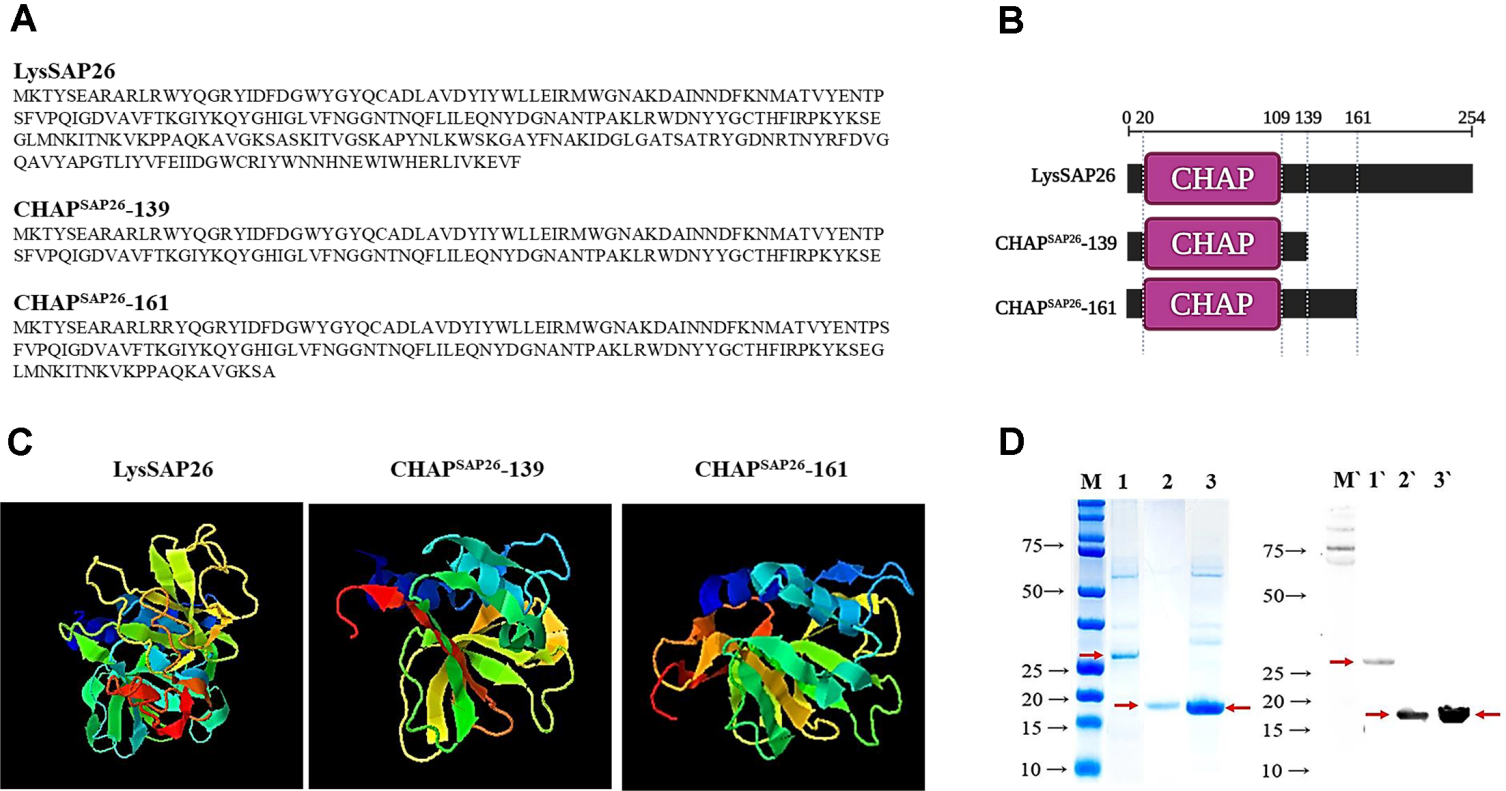
Amino acid sequences (A) schematic diagrams with CHAP domain (B) 3-D structures (C) and purified protein gel analyses (SDS-PAGE, left and Western blotting, right) of LysSAP26, CHAP^SAP26^-139, and CHAP^SAP26^-161 (D). Lane M is marked for the protein marker. Lanes 1 and 1' was LysSAP26, lanes 2 and 2' for CHAP^SAP26^- 139, and lanes 3 and 3' for CHAP^SAP26^-161. M and M' are molecular size markers.

To elucidate the physical, chemical, and structural characteristics of the CHAP^SAP26^-139 and CHAP^SAP26^-161 protein, a thorough bioinformatic examination was conducted (refer to Fig. 1). The alignment and validation of these amino acid sequences were executed using the BLASTP and Clustal Omega algorithms. Expasy’s ProtParam tool (https://web.expasy.org/protparam/) was used to predict various key properties, such as molecular weight, theoretical isoelectric point (pI), and extinction coefficient. In addition, the i-TASSER unified platform (https://seq2fun.dcmb.med.umich.edu/I-TASSER/) was used to analyze the three-dimensional structures and potential functional attributes of these proteins.

### Determination of the Antibacterial Activity of the Truncated Endolysins

Using a modified broth microdilution method in 96-well, round-bottomed microplates (SPL, Republic of Korea), the minimal inhibitory concentration (MIC) and MBC of the endolysins were determined according to the CLSI guidelines [36, 37]. Various concentrations (1–100 μg/ml) of proteins were added to the bacterial cells (10^6^ CFU/ml) and incubated for 18–24 h at 37°C. MIC was identified as the lowest endolysin concentration that completely inhibited bacterial growth. A 10-μl sample from each well of the MIC assay was transferred onto MHA plates to determine the MBC, followed by further incubation for 18–24 h to establish bactericidal activity. All assays were performed in triplicate to ensure reproducibility and reliability of the results.

### Time-Kill Assay of CHAP^SAP26^-161

A time-kill analysis was conducted to elucidate the time-sustained antibacterial efficacy of CHAP^SAP26^-161 against *A. baumannii* ATCC 17978 and *S. aureus* ATCC 25923 [29]. For this purpose, bacterial suspensions were prepared using the microdilution method, and CHAP^SAP26^-161 was added to achieve final concentrations of 10–50 μg/ml. The suspensions were incubated for 0, 2, 4, 6, 8, and 24 h, after which they were plated on LB agar to determine the CFUs. A dialysis buffer was used as a negative control. To ensure reproducibility of the results, the experiment was conducted twice independently.

### Effects of Temperature, pH, and Divalent Cations on the Antibacterial Activity of CHAP^SAP26^-161

A turbidity reduction assay assessed the effects of temperature, pH, and the presence of divalent ions on the antibacterial activity of CHAP^SAP26^-161. To assess the thermal stability of CHAP^SAP26^-161, it was pre-incubated at a broad temperature range (4°C to 60°C) for 2 h. To evaluate the enzymés pH stability, the endolysins' pH was adjusted to a range of pH 3 to 10 using HCl and NaOH and then incubated at 37°C for 2 h. Afterward, they were dialyzed in a neutral dialysis buffer (50 mM Tris-HCl, 500 mM NaCl, and 1 mM ZnCl_2_; pH 7.4). Wild type and mutant endolysins (concentration 10 to 50 mg/μl) which were pre-treated at various temperatures and pH levels were inoculated with target bacteria (A. baumanii ATCC17978 or *S. aureus* ATCC25923) prepared in a 96-well plate and incubated overnight at 37°C. Afterward, the optical density of each well at OD_600_ was measured. Furthermore, to determine the optimal cofactor for the endolysin, the antibacterial effect was analyzed as described above after adding 1 mM of each divalent ion (CaCl_2_, CuCl_2_, MgCl_2_, ZnCl_2_, and ZnSO_4_) or 10 mM of Ethylenediaminetetraacetic acid (EDTA) to the buffer. Statistical analyses were conducted to identify significant differences when compared with either the most active sample (*e.g.*, temperature and pH) or the blank control (*e.g.*, metal ion exposure and EDTA).

### Cytotoxicity of CHAP^SAP26^-161

Human lung epithelial cells (A549), which originate from adenocarcinoma, were used to evaluate the cytotoxic potential of CHAP^SAP26^-161. Cytotoxicity was assessed using the MTT assay (3-[4,5-methylthiazol-2-yl]-2,5-diphenyl-tetrazolium bromide, Amresco, USA), following the manufacturer’s protocol. Initially, A549 cells (1 × 10^5^ cells/well) were plated in a 24-well plate with RPMI medium and incubated overnight in a CO_2_ incubator to allow for cell attachment. Subsequently, the medium was replaced with fresh RPMI containing varying concentrations of CHAP^SAP26^-161 (ranging from 25 to 1,000 μg/ml). Under these conditions, the cells were incubated for an additional 24 h. Following this period, the medium was discarded and the cells were rinsed with phosphate-buffered saline (PBS). Each well then was added with 250 μl of MTT solution (0.5 mg/ml), and the cells were further incubated for 2 h. Finally, 250 μl of a solubilizing solution (90% isopropanol, 0.01% Triton X-100, and 0.01 N HCl) was added. The resultant color development, which is indicative of cell viability, was quantified at 570 nm using a VersaMax microplate reader (Molecular Devices, USA).

### Protection Efficacy of CHAP^SAP26^-161 in Mouse Model of *Acinetobacter baumannii* Infection

*A. baumannii* ATCC 17978 was used for the protection assay in a murine systemic infection model. Pathogen-free female BALB/c mice (6 weeks old, weight 16–19 g) were obtained from OrientBio (OrientBio, Republic of Korea). Neutropenic mice was induced by intraperitoneal injection of cyclophosphamide (150 mg/kg) on days -4 and -1 before bacterial injection. Systemic infection was induced via intraperitoneal injection of 200 μl (1 × 10^9^ CFU) of a log-phase bacterial inoculum. Mice were divided into six groups as follows (five mice/group):

● Group 1: Inactive control (200 μl PBS + 200 μl buffer A)

● Group 2: Infection control (200 μl *A. baumannii* + 200 μl buffer A)

● Group 3: CHAP^SAP26^-161 safety test (200 μl PBS + 50 μg/200 μl CHAP^SAP26^-161)

● Group 4: Positive control with LysSAP26 treatment (200 μl *A. baumannii* + 50 μg/200 μl LysSAP26)

● Group 5: CHAP^SAP26^-161 treatment (200 μl *A. baumannii* + 50 μg/200 μl CHAP^SAP26^-161)

Mice in each group were treated 30 min after infection, and postinfection survival was monitored once a day for 7 days following infection. All experimental animal procedures were approved by the Animal Care Committee of Kyungpook National University, with approval numbers 2022-0430.

### Statistical Analysis

Statistical analysis was conducted using OriginPro, applying one-way analysis of variance (ANOVA) followed by Tukey’s test for all pairwise comparisons (95% confidence interval) [38, 39]. Data are expressed as mean values with standard deviations, and statistical significance was set at a *p* value of < 0.01 [40].

## Results

### *In sillico* Analyses and Purification of LysSAP26 and Two Truncated Endolysins

We evaluated the structural characteristics and antibacterial activities of newly designed endolysin variants CHAP^SAP26^-139 and CHAP^SAP26^-161, created by truncating the C-terminus of LysSAP26 (Fig. 1A and 1B). When generating truncated mutants, the main focus was whether the proteins maintained their solubility after purification without aggregation or denaturation. Therefore, *in-silico* analysis using SWISS-MODEL from Expasy's ProtParam tool predicted that the cleavage sites at positions 139, 147 and 161 would exhibit solubility in the neutral to hydrophilic range. Recombinant proteins truncated at positions 139, 147, and 161 were produced, but truncation at 147 resulted in protein aggregation and insolubility. Therefore, CHAP^SAP26^-139 and CHAP^SAP26^-161 were compared with LysSAP26. The predicted isoelectric points (pI) of these proteins were 9.32 for CHAP^SAP26^-161 and 7.70 for CHAP^SAP26^-139. Structural modeling with I-TASSER suggested that LysSAP26 primarily forms helical structures interspersed with random coils (Fig. 1C), a typical characteristic of the CHAP domain-containing endolysins. CHAP^SAP26^-139 and CHAP^SAP26^-161 showed distinct structural differences compared to LysSAP26 (Fig. 1C). The shorter variant, CHAP^SAP26^-139, notably significantly reduced helical content at the truncated C-terminus, replaced mainly by coil structures, which has led to decreased structural stability or altered substrate binding. In contrast, while CHAP^SAP26^-161 retained a portion of its helical structure, but displayed changes in coil and helice distribution compared to LysSAP26.

LysSAP26 and the two truncated mutants were successfully expressed in *Escherichia coli*, as confirmed by SDS-PAGE, with molecular weights of 29.1 kDa for LysSAP26, 18.6 kDa for CHAP^SAP26^-161 and 16.2 kDa for CHAP^SAP26^-139 (Fig. 1D). SDS-PAGE and western blot analysis showed that the yield of CHAP^SAP26^-161 was highest among three proteins. The protein concentrations were 2.67 mg/l for LysSAP26, 17.32 mg/l for CHAP^SAP26^-161, and 6.33 mg/l for CHAP^SAP26^-139.

### Antibacterial Activities of LysSAP26 and Its Two Truncated Endolysins

The antibacterial activities of CHAP^SAP26^-161 and CHAP^SAP26^-139 were compared with that of LysSAP26 (Table 1). CHAP^SAP26^-161 showed a higher inhibitory activity than LysSAP26 against all bacterial species tested, whereas CHAP^SAP26^-139 did not. CHAP^SAP26^-161 revealed two-to five-fold lower MIC and MBC than LysSAP26. CHAP^SAP26^-161 showed the highest lytic activity against *A. baumannii* with an MBC of 5–10 μg/ml, followed by *S. aureus* with an MBC of 10–25 μg/ml, among the 12 types of bacterial species tested. Interestingly, CHAP^SAP26^-161 demonstrated lytic activity against *C. difficile*, an anaerobic Gram-positive bacteria with an MBC of 25–50 μg/ml (Table 1), but not against other anaerobic bacteria, such as Gram-positive *C. acnes* and Gram-negative *F. varium* (Table 1).

**Fig. 2:**
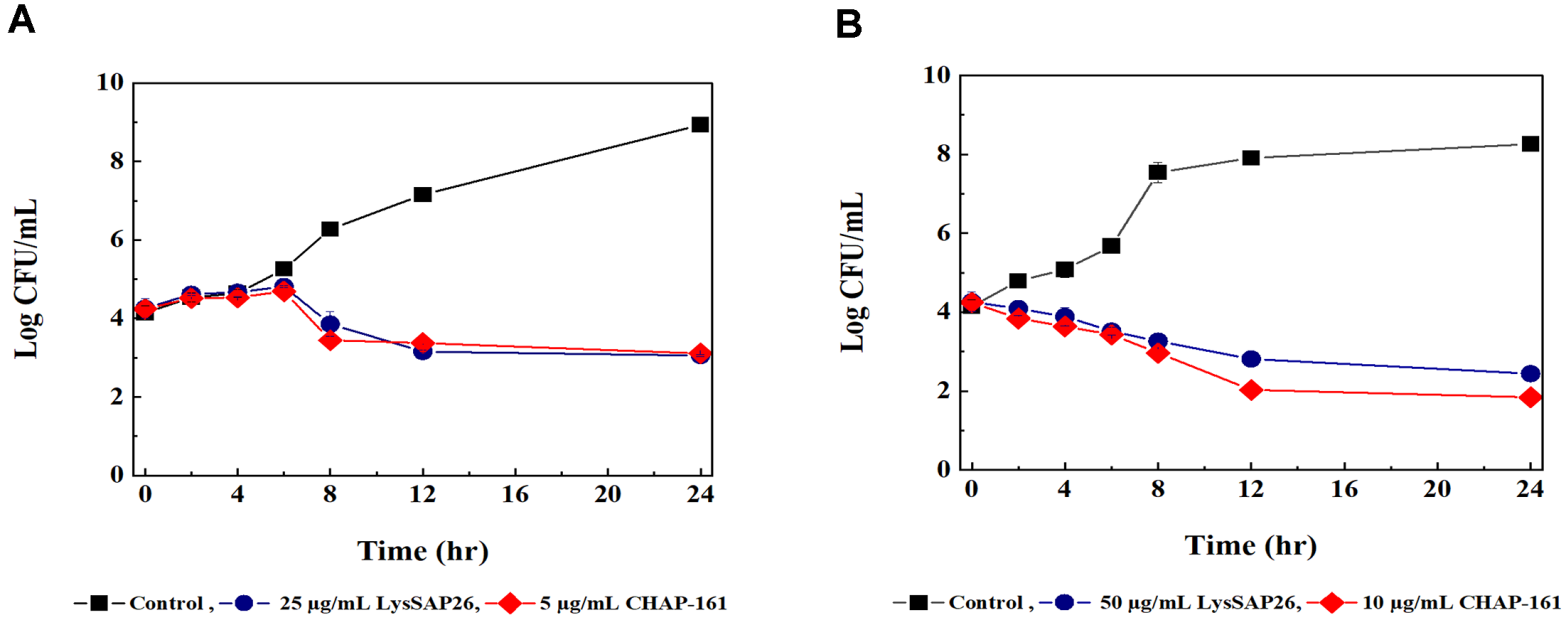
Time-kill assay results of LysSAP26 and CHAP^SAP26^-161 with *Acinetobacter baumannii* ATCC 17978 and (A) and *Staphylococcus aureus* ATCC 25923 (B).

Treatment with 25 μg/ml of LysSAP26 or 5 μg/ml of CHAP^SAP26^-161 reduced the number of CFU of *A. baumannii*, which began to occur after 6 h and was maintained until 24 h (Fig. 2A). When *S. aureus* was treated with 25 μg/ml of LysSAP26 or 10 μg/ml of CHAP^SAP26^-161, the number of CFU began to decrease immediately after treatment and was 2 log CFU lower than the initial value after 12 h (Fig. 2B).

The antibacterial efficacy of LysSAP26 and CHAP^SAP26^-161 against clinical MDR isolates was also compared. In total, 117 clinical isolates of *E. faecium*, *S. aureus*, *K. pneumoniae*, *A. baumannii*, *P. aeruginosa*, and *E. coli* were included in the susceptibility test, and the results are shown in Table 2. Similar to the results presented in Table 1, CHAP^SAP26^-161 showed two- to five-fold lower MIC and MBC than LysSAP26 (Table 2). CHAP^SAP26^-161 showed MICs and MBCs of 5–25 μg/ml against all 17 carbapenem-resistant *A. baumannii* isolates. The MIC values of CHAP^SAP26^-161 were 20 μg/ml for all 20 oxacillin-resistant *S. aureus* isolates, 25–50 μg/ml for 20 carbapenem-resistant *K. pneumonia*e isolates, and 25–50 μg/ml for 20 carbapenem-resistant *P. aeruginosa* isolates. Among20 carbapenem-resistant or cephalosporin-resistant *E. coli* isolates, MIC values were 50 μg/ml for 2 isolates and 25 μg/ml for 18 isolates. The MIC values of CHAP^SAP26^-161 against 20 vancomycin-resistant *E. faecium* isolates were 25 μg/ml (14 isolates) and 50 μg/ml (6 isolates) (Table 2).

### Effects of pH, Temperature, and Ions on CHAP^SAP26^-161 Activity

**Fig. 3:**
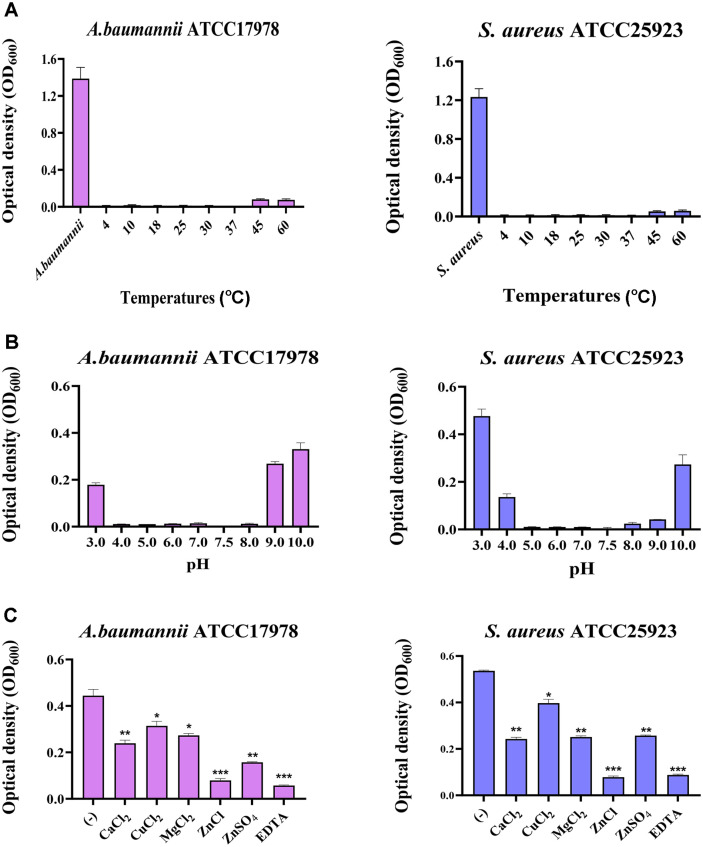
Effect of pH, temperature, and ions on bactericidal activity of CHAP^SAP26^-161 against *Acinetobacter baumannii* ATCC17978 and *Staphylococcus aureus* ATCC25923. (**A**) Temperature (**B**) pH (**C**) 1 mM ions (CaCl_2_, CuCl_2_, MgCl_2_, ZnCl_2_, and ZnSO_4_), and EDTA (10 mM). The Y axis shows optical density values at 600 nm of bacterial solution after the reactions under the different conditions. Statistically significant differences are indicated as *p < 0.1, **p < 0.001, and ***p < 0.001.

Fig. 3 demonstrates the effects of temperature, pH, and various ions on the enzymatic activity of CHAP^SAP26^-161. The antibacterial activity of CHAP^SAP26^-161 against *A. baumannii* and *S. aureus* were maintained at OD_600_ levels below 0.01 after incubation for one hour at temperatures ranging from 4°C to 37°C. However, at 60°C, the OD_600_ increased to between 0.06-0.086 (Fig. 3A). The antibacterial activity of CHAP^SAP26^-161 varied with pH against *A. baumannii* and *S. aureus*. For *A. baumannii*, the antibacterial effect was not affected by pH values between 4 and 8, maintaining OD_600_ between 0.01-0.015. In contrast, for *S. aureus*, effective antibacterial activity was observed at pH levels from 5 to 7.8, with OD_600_ remaining between 0.01-0.015 (Fig. 3B). The addition of ZnCl_2_ significantly enhanced the antibacterial activity of CHAP^SAP26^-161 compared to other divalent ions, lowering OD_600_ to below 0.09 (Fig. 3C). Upon the addition of EDTA at a concentration of 10 mM and after 18 h of treatment, the antibacterial effect observed resulted in OD_600_ values below 0.09 for both bacterial strains.

### Cytotoxicity of CHAP^SAP26^-161 and Its Protection Efficacy in *A. baumannii*-Infected Mouse Model

The MTT assay results showed that CHAP^SAP26^-161 did not affect the metabolic activity of A549 cells at concentrations from 25 μg/ml to 500 μg/ml, as evidenced by the over 90% cell survival rate. This indicates that CHAP^SAP26^-161 shows no cytotoxic effects on A549 cells up to a concentration of 500 μg/ml (Fig. S1).

**Fig. 4:**
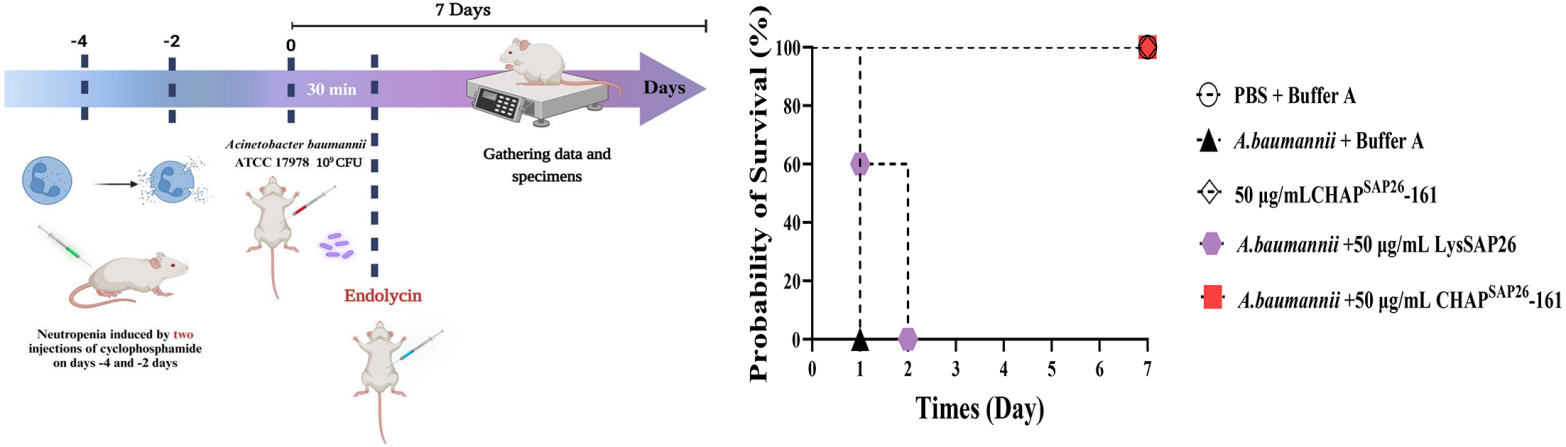
In vivo protection efficacy of CHAP^SAP26^-161 in mouse model infected with *Acinetobacter baumannii* ATCC 17978. Statistical significance was observed (*p < 0.01).

In vivo experiments with infection mouse model were performed to test the therapeutic effectiveness of CHAP^SAP26^-161 (Fig. 4). In the *A. baumannii* systemic infection model, neutropenic mice was induced and infected with *A. baumannii*, and treated with CHAP^SAP26^-161 or LysSAP26.

Although all mice were lethal on the first day of infection (Group 2), infected mice treated with 50 μg of CHAP^SAP26^-161 (Group 5) had a 100% survival rate over 7 days. In contrast, all infected mice treated with the same dose of LysSAP26 (Group 4) died within 2 days. Single CHAP^SAP26^-161 injection resulted in no fatal or adverse effects (Group 3) as did the inactive control (Group 1).

## Discussion

CHAP domains, which are integral components of bacterial amidases, autolysins, and bacteriophage-encoded peptidoglycan hydrolases, have been identified as critical functional protein regions in cleaving the molecular bridges that link peptidoglycan strands within bacterial cell walls, thereby facilitating cell lysis [41]. These domains are characteristic of a broader family of murein hydrolases, distinguished by Cys and His residues within their active sites, which are critical for their catalytic activity [3]. LysK consists of 495 amino acid residues. This endolysin is characterized by a CHAP domain spanning residues 35 to 160, an amidase-2 domain extending from residues 197 to 346, and an SH3b domain between residues 412 and 481 [42]. Horgan *et al*. showed that a truncated mutant of LysK [42], with deletions from residue 163 to the protein’s C-terminus, retained its lytic activity against methicillin-resistant *S. aureus* (MDR *S. aureus*). Two truncated mutants of LysSAP26, CHAP^SAP26^-139 and CHAP^SAP26^-161, were engineered by removing the C-terminal domain which is a potential cell binding module. This decision was guided by prior studies showing the significant role of the CHAP domain in endolysins, such as LysK, and the impact of C-terminal truncations on bactericidal activity, as observed in LysSAP33 [21, 29, 43, 44]. The purification processes for CHAP^SAP26^-139 and CHAP^SAP26^-161 demonstrated superior outcomes compared with LysSAP26, with significant reductions in undesirable protein contamination and enhancements in protein yields at the final purification stage. Notably, CHAP^SAP26^-161 exhibited the highest yield among the proteins tested, suggesting that the absence of the C-terminal domain contributes to its solubility and stability in the purification buffer. Moreover, the purity of CHAP^SAP26^-161 was further enhanced via repeated affinity or size exclusion chromatography. Among the two truncated LysSAP26 mutants, CHAP^SAP26^-139 showed limited or weaker activity against all tested bacterial species than the wild-type and CHAP^SAP26^-161 proteins. These findings indicate that the amino acid residues from 140 to 161 are crucial for the enzymatic activity of CHAP. Notably, CHAP^SAP26^-161 revealed bactericidal activities that were 2.5–5-fold greater against *S. aureus* and *A. baumannii* than the wild-type protein, according to protein weight. Altogether, we produced a smaller CHAP-containing protein possessing similar or better antibacterial activity and protein purification yields than LysSAP26.

The phage Twort endolysin (PlyTW) is composed of three distinct domains: a CHAP domain, an amidase-2 domain, and an SH3b-5 cell binding domain (CBD) [20]. Particularly, the isolated CHAP domain is capable of lysing *S. aureus* in-vivo, with the absence of CBD resulting in a 10-fold decline in enzymatic efficacy. Yu *et al*. engineered a truncated variant of LysSAP33 (residues 1–156; CHAP-156), which shares identity with LysSAP26, albeit originating from a different phage, and observed a significant diminution in lytic efficiency upon removal of the C-terminal domain, highlighting its importance [43]. Contrary to the findings of Yu *et al*., our research postulates that CHAP proteins lacking the C-terminal domain, exemplified by CHAP^SAP26^-161, show superior antibacterial ability against a broader spectrum of bacterial strains compared with the original enzyme. In addition, our results in conjunction with those of Yu *et al*. suggest that the segment comprising residues 1–161 of LysSAP26 represents the minimal functional unit capable of achieving optimal antibacterial activity upon C-terminal truncation.

The antibacterial activity of CHAP^SAP26^-161 against *A. baumannii* and *S. aureus* sustained >95% of its activity after 1 h of incubation at 4°C–37°C; however, it decreased by approximately 15%–20% at 60°C (Fig. 3A). The effect of pH on the antibacterial activity of CHAP^SAP26^-161 against *A. baumannii* and *S. aureus* varied. The antibacterial activity of CHAP^SAP26^-161 against *A. baumannii* remained unaffected by pH between 4 and 8, but that against *S. aureus* was reduced under pH 4 (Fig. 3B). CHAP^SAP26^-161 exhibited antibacterial activities that were nearly comparable to those of LysSAP26, albeit under conditions of strong acidity and alkalinity. This indicates that the C-terminal domain may be crucial in maintaining protein stability under extreme pH conditions. Interestingly, the addition of ZnCl_2_ dramatically increased the antibacterial activity of CHAP^SAP26^-161 compared with those with other divalent ions (Fig. 3C). The wild-type protein and CHAP^SAP26^-161 protein required the addition of EDTA or the presence of ZnCl_2_ to enhance enzymatic activity. EDTA damages bacteria by chelating bacterial ions, thereby enhancing the antibacterial effect of the endolysins. In contrast, when EDTA is not used, and bacteria are not damaged, the presence of cations enhances the antibacterial effect of the endolysins. Among various cations, the addition of ZnCl_2_ was found to be the most effective. Conversely, PlyTW demonstrated increased antibacterial activity in the presence of Ca^2+^ ions but inhibited activity when exposed to zinc ions or EDTA [20].

Our in-vivo experiments conducted on mice to evaluate the antibacterial efficacy of CHAP^SAP26^-161 against *A. baumannii* suggest that CHAP^SAP26^-161 possesses considerable therapeutic potential (Fig. 4). In a neutropenic mouse model infected with *A. baumannii*, treatment with CHAP^SAP26^-161 resulted in a 100% survival rate, significantly improving compared with the LysSAP26 treatment group (Fig. 4 B). Notably, the survival time in the CHAP^SAP26^-161-treated group was substantially enhanced relative to that in the infection control group. This study provides a deeper understanding of the therapeutic effects of CHAP^SAP26^-161 and offers crucial foundational data for future research on developing antibacterial protein architecture.

Considering the use of these proteins for clinical or veterinary treatments, the aforementioned ionic conditions must be considered. Preliminary findings from our pilot study indicate that CHAP^SAP26^-161 has potential as an effective antibacterial agent against pathogens such as *Bacillus megaterium*, *Bacillus muralis*, *Corynebacterium striatum*, and *E. faecium*, which are known to contaminate catheters in clinical environments [35].

In summary, our investigation has led to the development of a truncated CHAP-containing protein variant, CHAP^SAP26^-161, which exhibits antibacterial activity and protein purification yields at par with or exceeding those of wild-type LysSAP26. To the best of our knowledge, this is the first report on the CHAP^SAP26^-161 protein demonstrating antibacterial activity against *C. difficile*, in addition to its efficacy against methicillin-resistant *S. aureus* (MDR *S. aureus*) and carbapenem-resistant *A. baumannii*. Given the significant challenge in generating resistant mutants to endolysins, CHAP^SAP26^-161 has emerged as a potential antibacterial candidate for combating drug-resistant bacterial infections.

## Supplemental Materials

Supplementary data for this paper are available on-line only at http://jmb.or.kr.



## Figures and Tables

**Table 1:** Antibacterial activity of LysSAP26, CHAP^SAP26^-161, and CHAP^SAP26^-139 against 19 strains of 11 bacterial species.

Bacterial strains	LysSAP26 (μM)		CHAP^SAP26^-139 (μg/ml)		CHAP^SAP26^-161 (μg/ml)	
	MIC^1^	MBC^2^	MIC	MBC	MIC	MBC
*Staphylococcus aureus*						
*S. aureus* ATCC 25923	25	25	>100	>100	10	10
*S. aureus* ATCC 29513	50	50	>100	>100	25	25
*S. aureus* ATCC 33591	50	50	>100	>100	25	25
*Staphylococcus epidermidis* clinical isolate KBN10P014768	>75	>75	>100	>100	75	75
*Enterococcus faecalis* ATCC 29212	50	75	>100	>100	25	25
*E. faecium* clinical isolate KBN10P02068	50	75	>100	>100	25	25
*Acinetobacter baumannii*						
*A. baumannii* ATCC 17978	25	25	75	75	5	5
*A. baumannii* ATCC 19606	25	25	100	100	10	10
*Klebsiella pneumoniae* KCTC 2208	50	50	>100	>100	25	25
*Pseudomonas aeruginosa*	50	50	>100	>100	25	25
*Clostridioides difficile*						
*C. difficile* KCTC 5009	50	50	>100	>100	25	25
*C. difficile* clinical isolate KBN10P03654	75	75	>100	>100	25	25
*C. difficile* clinical isolate KBN10P03780	75	75	>100	>100	50	50
*C. difficile* clinical isolate KBN10P03783	75	75	>100	>100	50	50
*Cutibacterium acnes* ATCC 6919	>100	>100	>100	>100	>100	>100
*Campylobacter jejuni*						
*C. jejuni* KCTC 5327	>100	>100	>100	>100	>100	>100
*C. jejuni* ATCC 33291	>100	>100	>100	>100	>100	>100
*Fusobacterium varium*						
*F. varium* clinical isolate B2-O-100	>100	>100	>100	>100	>100	>100
*F. varium* clinical isolate B2-F-100	>100	>100	>100	>100	>100	>100

^1^MIC: Minimum inhibitory concentration

^2^MBC: Minimum bactericidal concentration

**Table 2:** Antibacterial activity of LysSAP26 and CHAP^SAP26^-161 against MDR clinical isolates of ESKAPE pathogens.

Bacteria from KNUHCCP^1^	No. of isolates tested	LysSAP26 (μg/ml)				CHAP^SAP26^-161 (μg/ml)			
		MIC^2^		MBC^3^		MIC		MBC	
		MIC_50_^4^	MIC_90_^5^	MBC_50_^6^	MBC_90_^7^	MIC_50_	MIC_90_	MBC_50_	MBC_90_
*A. baumannii*	17	10	50	10	50	5	25	5	25
*S. aureus*	20	25	75	25	75	5	25	5	25
*K. pneumoniae*	20	50	75	50	75	25	50	25	50
*P. aeruginosa*	20	50	75	50	75	25	50	25	50
*E. faecium*	20	50	75	50	75	25	50	25	50
*E. coli*	20	50	75	50	75	25	50	25	50

^1^KNUHCCP: Kyungpook national university hospital culture collection for pathogens

^2^MIC: Minimum inhibitory concentration

^3^MBC: Minimum bactericidal concentration

^4^MIC_50_: Minimum inhibitory concentration that inhibits visible growth of 50% of the isolates

^5^MIC_90_: Minimum inhibitory concentration that inhibits visible growth of 90% of the isolates

^6^MBC_50_: Minimal bactericidal concentration that kills bacteria of 50% of the isolates

^7^MBC_90_: minimal bactericidal concentration that kills bacteria of 90% of the isolates
