# Supplementary figures and images for: A Novel Truncated CHAP Modular Endolysin, CHAPSAP26-161, That Lyses Staphylococcus aureus, Acinetobacter baumannii, and Clostridioides difficile, and Exhibits Therapeutic Effects in a Mouse Model of A. baumannii Infection
Source: J Microbiol Biotechnol. 2024 Jun 17;34(8):1718–26. doi: 10.4014/jmb.2402.02042 (PMC11380504; doi:10.4014/jmb.2402.02042)

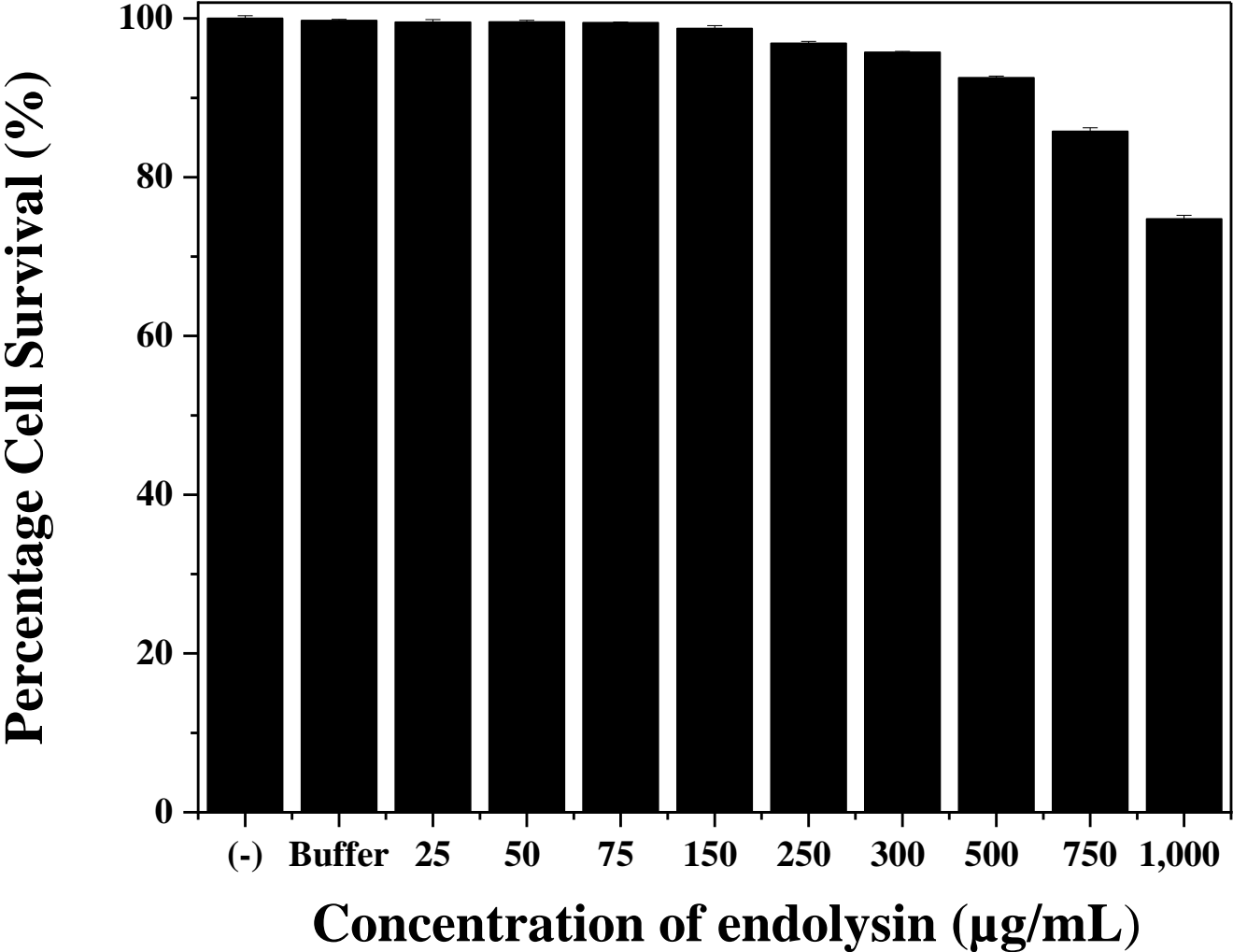

Supplement: Supplementary file 1 [file jmb-34-8-1718-supple.pdf]
